# Special Issue “Biodegradation and Environmental Microbiomes”: Editorial

**DOI:** 10.3390/microorganisms11051253

**Published:** 2023-05-10

**Authors:** Shuang-Jiang Liu

**Affiliations:** 1 State Key Laboratory of Microbial Technology, Shandong University, Qingdao 266237, China; liusj@sdu.edu.cn or liusj@im.ac.cn; 2 State Key Laboratory of Microbial Resources and Environmental Microbiology Research Center, Institute of Microbiology, Chinese Academy of Sciences, Beijing 100101, China

The Earth is unique, and we as human beings rely on its air, water, and land. Industrialization has improved our daily life at the cost of nature resources and environmental quality. Environmental pollution has reduced social sustainable development, and even caused it to adversely affect our body [1]; new technologies are needed to address these challenges. Biodegradation and bioremediation are promising technologies that can return humanity to a sustainable development [2]. Microbes in total and their interactions with their dwelling environment and/or host, which is the core concept of microbiomes [3], are the main driving force for biodegradation and bioremediation of chemically synthesized compounds or natural products generated from malfunctioning ecosystems. This Special Issue “Biodegradation and Environmental Microbiomes” has collected 17 papers [4,5,6,7,8,9,10,11,12,13,14,15,16,17,18,19,20] that cover diverse researches in this field of environmental microbiology and bioremediation.

Of the 17 papers, 3 are reviews. As the first reader, the editor is happy to read the two reviews [4,5] from Yuan’s lab, a pioneering group of scientists in synthetic biology, that summarize and prospect the state-of-art of biodegradation of plastics, including polyethylene terephthalate (PET), polyethylene (PE), polypropylene (PP), and polystyrene (PS), and particularly emphasize the great potential of synthetic biology tools for construction of artificial microbial consortia or artificial synthetic microbiomes for the efficient degradation of plastics. This Special Issue also discloses new microbial resources, in addition to the microbial resources summarized in reviews [4,5], that are key to efficient removal of naturally generated but disgusting materials from malfunctioning ecosystems [7,8] or chemically synthesized compounds such as decabromodiphenyl ether [10], tetracycline [13], and sulfamethoxazole [14]. Ma et al. [8] screened out bacterial strains from composting sites for highly efficient deodorization and suppression of the odor gas of pig manure, which has high application value for the control of odor pollution. Yu et al. [14] reported two bacteria, *Paenarthrobacter ureafaciens* and *Pseudomonas koreensis*, that work together and improve the degradation of sulfamethoxazole. In this Special Issue, Ngara et al. [11] provided a Biological Nitrogen Removal Database (BNRdb) curated resources of 23,308 microbial strains, 46 gene families, 24 enzymes, 18 reactions, 301 BNR treatment datasets, 860 BNR-associated next-generation sequencing datasets, and 6 common BNR bioreactor systems, a user-friendly interface enabling interactive data browsing.

The regulation of biofilm formation, chemotaxis towards pollutants, and the microbiome compositions and assembling are fundamental questions that have been extensively studied for decades, but new understandings are continuously emerging as reported in this Special Issue [9,12,15,17]. Wang et al. [9] reported that the chemotaxis regulator FlmD of *Comamonas testosteroni* modulates biofilm formation in a *c*-*di*-GMP independent way. Zhou et al. [17] observed that PAHs-degrading *Sphingomonas* species was the core and keystone microbe of the PAHs-polluted soil microbiomes, and further proposed that the abundant flagellar assembly and bacterial chemotaxis genes in the assembled genome of this *Sphingomonas* species exhibited potential for competitive advantage in the soil microbiomes. Duan et al. [15] reported that inoculation of *Lysinibacillus* sp. and *Penicillium oxalicum* (LFPO group) effectively altered the microbial communities for composting of wheat straw and cow manure, accelerated organic matter and lignocellulose degradation, and contributed to longer thermophilic fermentation and lower microbial diversity in the LFPO treatment compared to the control group. Interestingly, the paper by Hu et al. revealed a core microbiome of tobacco leaves nation-wide in China that contains only 14 operational taxonomic units (OTUs) of nine species, and the function of this core microbiome is related to processes including nitrogen fixation, detoxification of diverse pollutants, and heavy-metal reduction. The leaf microbiome (4473 OTUs in total, representing 1234 species) was affected by local environments but did not exhibit obvious relationships to single ecological factors (e.g., temperature, precipitation) [12].

At the time of this writing, papers published in this Species Issue were viewed 29,636 and cited for 65 times based on statistics of the Microorganisms website. As the guest editor, I offer all authors and reviewers my heartful thanks and gratitude for their contributions of research and time that made this Special Issue successful. I would also like to thank my co-editors, Prof. Dr. Hongzhi Tang, Prof. Dr. Jiandong Jiang, and Prof. Dr. Xiaolei Wu, for their efforts on this Special Issue. Although this Special Issue is closed, “Biodegradation and Environmental Microbiomes” remains as a topic platform, and continues to serve authors for sharing new knowledge in the related fields.
